# Within-trial cost-effectiveness of lifestyle intervention using a 3-tier shared care approach for pregnancy outcomes in Chinese women with gestational diabetes

**DOI:** 10.1371/journal.pone.0237738

**Published:** 2020-08-20

**Authors:** Weiqin Li, Cuiping Zhang, Junhong Leng, Ping Shao, Huiguang Tian, Fuxia Zhang, Ling Dong, Zhijie Yu, Juliana C. N. Chan, Gang Hu, Ping Zhang, Xilin Yang

**Affiliations:** 1 Tianjin Women and Children’s Health Centre, Tianjin, China; 2 Department of Epidemiology, Capital Institute of Pediatrics, Beijing, China; 3 Graduate School of Peking Union Medical College, Beijing, China; 4 Population Cancer Research Program and Department of Pediatrics, Dalhousie University, Halifax, Canada; 5 Department of Medicine and Therapeutics, Hong Kong Institute of Diabetes and Obesity and The Chinese University of Hong Kong-Prince of Wales Hospital-International Diabetes Federation Centre of Education, Hong Kong SAR, China; 6 Chronic Disease Epidemiology Laboratory, Pennington Biomedical Research Center, Baton Rouge, Louisiana, United States of America; 7 Division of Diabetes Translation, Centers for Disease Control and Prevention, Atlanta, Georgia, United States of America; 8 Department of Epidemiology and Biostatistics, School of Public Health, Tianjin Medical University, Tianjin, China; Texas A&M University College Station, UNITED STATES

## Abstract

This study assessed within-trial cost-effectiveness of a shared care program (SC, n = 339) for pregnancy outcomes compared to usual care (UC, n = 361), as implemented in a randomized trial of Chinese women with gestational diabetes (GDM). SC consisted of an individualized dietary advice and physical activity counseling program. The UC was a one-time group education program. The effectiveness was measured by number needed to treat (NNT) to prevent one macrosomia/large for gestational age (LGA) infant. The cost-effectiveness was measured by incremental cost-effectiveness ratio in terms of cost (2012 Chinese Yuan/US dollar) per case of macrosomia and LGA prevented. The study took both a health care system and a societal perspective. This study found that the NNT was 16/14 for macrosomia/LGA. The incremental cost for treating a pregnant woman was ¥1,877 ($298) from a health care system perspective and ¥2,056 ($327) from a societal perspective. The cost of preventing a case of macrosomia/LGA from the two corresponding perspectives were ¥30,032/¥26,278 ($4,775/$4,178) and ¥32,896/¥28,784 ($5,230/$4,577), respectively. Considering the potential severe adverse health and economic consequences of a macrosomia/LGA infant, our findings suggest that implementing this lifestyle intervention for women with GDM is an efficient use of health care resources.

## Introduction

Gestational diabetes mellitus (GDM), defined as diagnosis of hyperglycemia of any degree during pregnancy, is associated with adverse pregnancy outcomes including macrosomia [1], preterm birth [1, 2], shoulder dystocia, birth trauma and neonatal morbidities [3–5]. GDM also predisposes offspring born to women who had GDM during the index pregnancy to higher risk of childhood obesity [6], and the mothers themselves to higher risk of developing diabetes during their lifetime [7, 8].

Lifestyle intervention is effective in improving pregnancy outcomes among women with GDM [9, 10]. The Australian Carbohydrate Intolerance Study in Pregnant Women (ACHOIS) reported that intensive lifestyle intervention reduced the rate of serious perinatal complications, defined as death, shoulder dystocia, bone fracture, and nerve palsy, macrosomia and preeclampsia among women with GDM [9]. GDM in this study was defined by the World Health Organization’s criteria (fasting plasma glucose levels ≥7.8 mmol/l, or 2-hour plasma glucose levels between 7.8 mmol/l and 11.1 mmol/l) [11], based on a 75-gram 2-hour oral glucose tolerance test (OGTT). Another multicenter randomized trial reported that intensive lifestyle intervention reduced the rates of macrosomia [10], shoulder dystocia and pregnancy-induced hypertension (PIH) [10] in women with mild GDM, defined by the Fourth International Workshop-Conference on GDM’s criteria (a fasting glucose level < 5.3 mmol/l and one glucose measurement that exceeds the following thresholds: 1-hour, 10.0 mmol/l; 2-hour, 8.6 mmol/l; and 3-hour, 7.8 mmol/l) [12], based on a 100-gram 3-hour OGTT.

We conducted a randomized trial of Chinese women with GDM using the International Association of Diabetes and Pregnancy Study Group’s (IADPSG) criteria (fasting plasma glucose levels ≥5.1 mmol/l, or 1-hour plasma glucose levels ≥10.0 mmol/l, or 2-hour plasma glucose levels ≥8.5 mmol/l, based on a 75-gram 2-hour OGTT) [13]. The primary objective of the study was to test if lifestyle intervention delivered in the 3-tier prenatal care system (SC), as compared with usual antenatal care (UC), could improve pregnancy outcomes among Chinese women with GDM. The secondary objective was to assess the cost-effectiveness of the lifestyle intervention program if the program was effective. We previously reported that the lifestyle intervention program reduced the risk of macrosomia and large for gestational age (LGA) [14]. Here we report the results of the cost-effectiveness of the intervention as implemented in the trial.

## Materials and methods

### Brief description of the trial

Details of the trial including background, methods, study outcomes and research settings were described elsewhere [14]. Briefly, between December 2010 and October 2012, 19,847 pregnant women who were between 24 and 28 weeks of gestation underwent a 50-gram 1-hour glucose challenge test (GCT) at primary hospitals within the 3-tier’s health care network in the six central urban districts of Tianjin, China. The 3-tier network prenatal care system consists of more than 300 primary hospitals throughout the city (first level), 16 district-level Women and Children’s Health Centers (WCHC), more than 50 secondary obstetric hospitals (second level), 1 city-level WCHC (TWCHC) and 7 tertiary obstetric hospitals (third level). Structured prenatal care was delivered within the network. First, all pregnant women in the city were registered at a primary hospital and received their routine prenatal care until the 32^nd^ gestational week. Then they were referred to one of the secondary or tertiary obstetric hospitals for the remainder of their prenatal care. If women experienced pregnancy complications, they were referred to third-level treatment facilities.

The GCT test identified 2921 women with plasma glucose (PG) values of ≥7.8 mmol/L. A following 75-gram OGTT test was performed in these women and 1,440 of them were diagnosed with GDM using the IADPSG’s criteria [14]. Of the 1440 women with GDM, the 948 who agreed to participate in the study and did not meet any of the exclusion criteria were randomly assigned to either the shared care (SC) group (n = 474) or the usual care (UC) group (n = 474). However, the intervention effect on 242 participants might have been contaminated due to (1) unavailability of separate spaces for the intervention and control groups at the central intervention site due to building renovation; and (2) the staff at this study site did not follow data collection procedures as specified in the study protocol (i.e., the same staff members collected data for both study groups due to insufficient manpower). Therefore, we excluded those 242 participants from the main analysis [14]. In addition, six study participants who chose to deliver their infants at hospitals outside Tianjin were excluded. Thus, 700 study participants were included in the final analysis: 339 from the SC and 361 from the UC. A simple randomization procedure without replacement (i.e., by the time sequence of visits to the clinic and a list of priori computer-generated random assignment status by X.Y.) was used to perform the random assignment by the intervention team members [14]. The study flow diagram (S1 Fig) shows the details. Major demographic and clinical information of the study participants are listed in S1 Table. There were no significant differences between the two groups for the baseline clinical and biochemical characteristics. A more detailed description of the study participants can be found in our previous study [14]. The RCT was registered at Clinicaltrials.gov (NCT01565564). Ethics approval of the original study was granted by the Human Subjects Committee of the Tianjin Women’s and Children’s Health Center, and all participants provided written informed consent.

Women in the UC group were offered one hospital-based group diabetes education session lasting for 30–40 minutes with a focus on diet and physical activity. In addition to UC, the SC group was offered structured intensive lifestyle intervention, including (1) one group general diabetes/GDM counseling and education materials at enrollment, (2) two detailed individualized consultations from trained doctors and laboratory testing at the time of the 30^th^ and 34^th^ gestational weeks, (3) three health education sessions with trained nurses at the time of the 27^th^, 29^th^ and 33^th^ gestational weeks, and (4) regular self-monitoring of blood glucose (SMBG) [14]. The dietary advice and physical activity counseling included recommendations on a tailored diet and a minimum 30 minutes daily of light/moderate physical activity such as walking. The diet recommendation was based on pre-pregnancy body mass index (BMI), i.e., 35 kcal per day for one kilogram of body weight (35 kcal/day/kg) for women with BMI at <18.5 kg/m^2^; 30–35 kcal/day/kg for women with BMI at 18.5–23.9 kg/m^2^; 25–30 kcal/day/kg for women with BMI at 24.0–27.9 kg/m^2^; 25 kcal/day/kg for women with BMI at 28.0 kg/m^2^ and more. In addition, the SC group was offered a free glucose meter with a memory function and free tests trips. The SC participants were also asked to monitor blood glucose (SMBG) 4 times a day for the initial two weeks and then daily at different time points (alternating between pre-breakfast and 2 hours after each of the three meals) until the end of pregnancy. Glycemic control goals were set at ≥3.5-≤5.1 mmol/l for fasting capillary blood glucose and ≤7.0 mmol/l for 2-hour postprandial capillary blood glucose up to the 36^th^ gestational week and ≤8.0 mmol/l from the 36^th^ week onwards. If blood glucose values exceeded the target values two times or more during a 2-week interval or the 2-hour postprandial capillary blood glucose exceeded 9.0 mmol/l once during a 1-week period, insulin therapy would be recommended by the intervention team and started by a senior obstetrician. The obstetric care was the same for both study groups.

### Cost-effectiveness analysis

We measured the cost-effectiveness of this lifestyle intervention using incremental cost-effectiveness ratio (ICER), which was calculated as the incremental cost divided by incremental effectiveness. The incremental cost is the net cost between the SC and UC, and the incremental effectiveness was the net effectiveness between the two study groups. The study took two perspectives: a health system perspective, which considered the direct medical cost only; and a societal perspective, which considered direct medical cost, direct non-medical cost and indirect cost. As we performed a within-trial analysis, our analytical time horizon was from randomization to postnatal hospital discharge of the mother and baby. Analyses were performed on an intention to treat basis. All costs were reported in Chinese yuan (CNY, ¥) (1 CNY = 0.159 US dollars, 31 December 2012).

### Effectiveness of the intervention

We measured the effectiveness of the intervention using the number needed to treat (NNT) for two health outcomes: macrosomia and LGA. Macrosomia was defined as birth weight ≥4,000 gram. LGA was defined by birth weight above the gender- and gestational age- specific 90^th^ percentile using the Tianjin local references. NNT was calculated as the inverse of the absolute risk reduction of macrosomia/LGA.

### Direct medical costs

Direct medical costs included the intervention costs and the routine surveillance, obstetric and neonatal complications attributable to GDM. The costs associated with various glucose tests to identify women with GDM were the same for both the SC and UC groups. These tests included a GCT at primary hospitals, an OGTT between the 24^th^ and 28^th^ gestational weeks. The cost associated with the initial hospital-based group diabetes education session upon diagnosis of GDM was also the same for both groups. Additional costs for the SC group included (1) one group general diabetes/GDM counseling and education materials at enrollment, (2) two detailed individualized consultations from trained doctors and laboratory testing at the time of the 30^th^ and 34^th^ gestational weeks, (3) three health education sessions with trained nurses at the time of the 27^th^, 29^th^ and 33^th^ gestational weeks, and (4) SMBG. All women in the SC group were offered a free glucose meter with a memory function and free test strips. Similar to the SC women, additional costs for the UC group included costs associated with a simple diabetes/GDM consultation and laboratory testing at the 34^th^ gestational. The specific cost items included are listed in Table 1. Itemized cost information for each study participant was recorded during the trial period.

**Table 1:** Intervention costs of a lifestyle intervention program for women with gestational diabetes in China, per study participant, by study arm.

Intervention phase	Shared care (n = 339, CNY, ¥)	Usual care (n = 361 CNY, ¥,)	Incremental cost (CNY, ¥,)	Data Sources
*Screening and diagnosis*: *24-28*^*th*^ *gestational week*				
GCT	15	15	0	MI
OGTT	120	120	0	MI
Plasma insulin	100	100	0	IDF
*Hospital-based education session upon diagnosis of GDM*	24	24	0	MI
*Enrollment and first intervention*				
Group counseling	50	0	50	IDF
Education materials	10	0	10	IDF
*Individual consultations*: *30*^*th*^ *and 34*^*th*^ *gestational week*				
Antenatal visit to Obstetricians	22	9	13	IDF
Laboratory testing during 30^th^ gestational week	19	0	19	IDF
Laboratory testing during 34^th^ gestational week	126	98	28	IDF
Nutrition analysis and diet consultation	73	0	73	IDF
Individual consultation	73	12	61	IDF
*Health education session*: *27*^*th*^, *29*^*th*^ *and 33*^*rd*^ *gestational week*	120	0	120	IDF
*Regular self-monitoring of blood glucose*:				
Glucose meters	380	0	380	IDF
Glucose test strips	200	0	200	IDF
*Insulin therapy in pregnancy*	12	3	9	IDF
**Total**	**1,344**	**381**	**963**	**MI or IDF**

All costs were reported in Chinese yuan (CNY, ¥) (1 CNY: 0.159 USD, 31 December 2012).

MI, Maternity insurance. IDF, International Diabetes Federation.

Costs for routine surveillance, obstetric and neonatal complications attributable to GDM were collected from the electronic database *Pregnant Women Health Records* and also from a questionnaire survey at 4–6 weeks after delivery, regardless of the random group assignment. Once women were diagnosed with GDM, they received a hyperglycemia review one week later. Women with GDM were automatically categorized as having a high-risk pregnancy which triggered multidisciplinary clinical management. The management involved clinical review, fetal surveillance and medications if needed at primary hospitals before the 32^nd^ gestational week, and referral to secondary or tertiary hospitals thereafter for continued care until delivery. Both costs of hospitalizations before and during delivery for obstetric and neonatal complications attributable to GDM were included in the analysis. Obstetric complications included poor metabolic control, pre-eclampsia, fetal macrosomia or fetal growth retardation, labor-induction and caesarean delivery. Neonatal complications included hypoglycemia, respiratory distress and other complications, and admission to the neonatal nursery (S2 Table).

### Direct non-medical costs and indirect costs

The cost items included in this category were: the travel expenses and time costs related to the use of health services; food costs associated with dietary changes; time lost due to morbidity and outpatient treatments; and overhead charges such as rental, utilities and maintenance costs for the office space. These costs were obtained via a questionnaire at 4–6 weeks after delivery, as listed in Table 2.

**Table 2:** Direct non-medical and indirect costs associated with a lifestyle intervention program for women with gestational diabetes in China, per study participant, by study arm.

	Shared care (n = 339, CNY, ¥)	Usual care (n = 361 CNY, ¥,)	Incremental cost (CNY, ¥,)	Sources
Travel to follow-up visits	305	283	22	IP
Costs of lower glycemic index snacks	1,095	924	171	IP
Time lost due to outpatient services	1,219	1,130	89	IP
Time lost due to morbidity	425	598	-173	IP
Overhead charges	140	70	70	IDF
**Total**	**3,184**	**3,005**	**179**	**IP or IDF**

All costs were reported in Chinese yuan (CNY, ¥) (1 CNY: 0.159 USD, 31 December 2012).

IP, Individual payment. IDF, International Diabetes Federation.

### Sensitivity analysis

The first sensitivity analysis was to examine how cost-effectiveness of the intervention would change by excluding data for the 242 women (130 in SC and 112 in UC) for whom the intervention effect could be contaminated as described earlier. The second was to examine how the result would change if the cost of implementing the intervention was higher in different settings by doubling intervention cost.

## Results

SC significantly reduced the rates of macrosomia [SC group vs. UC group: 11.2% (38/339) vs. 17.5% (63/361), p = 0.019] and LGA [SC group vs. UC group: 13.0% (44/339) vs. 19.9% (72/361), p = 0.013] in comparison to UC. The NNT for the intervention was 16 to prevent one macrosomia, and 14 to prevent one LGA.

The estimated cost of various cost components per study participant by study group are described in Tables 1–3 and S2 Table, including intervention cost in Table 1, the non-medical and indirect costs in Table 2, the total cost in Table 3, and costs associated with routine surveillance, obstetric and neonatal complications in S2 Table. The direct medical costs per study participant in the SC and UC groups were ¥10,892 and ¥9,015, respectively, which included intervention costs (¥1,344 and ¥381) and costs of routine surveillance, obstetric and neonatal complications attributable to GDM (¥9,549 and ¥8,634). The direct non-medical and indirect costs per subject were ¥3,187 and ¥3,005 in the SC and UC groups, respectively.

**Table 3:** Total costs of a lifestyle intervention program for women with gestational diabetes in China, per study participant, by study arm.

	Shared care (n = 339, CNY, ¥)	Usual care (n = 361 CNY, ¥,)	Incremental cost (CNY, ¥,)
*Direct medical costs per subject*			
Intervention costs	1,344	381	963
Routine surveillance, obstetric and neonatal complications attributable to GDM	9,549	8,634	915
Subtotal	10,892	9,015	1,877
*Direct non-medical and indirect costs per subject*	3,184	3,005	179
Total cost per subject			
Health system perspective*	10,892	9,015	1,877
Social perspective*	14,076	12,020	2,056

All costs were reported in Chinese yuan (CNY, ¥) (1 CNY: 0.159 USD, 31 December 2012).

* Health system perspective cost only included the direct medical costs; societal perspective cost included direct medical cost, direct non-medical cost, and indirect cost.

From the health system perspective, the total cost per study participant was ¥10,892 in the SC group and ¥9,015 in the UC group, and the incremental cost was ¥1,877. From the societal perspective, the cost per study participant in the SC/UC groups was ¥14,076/¥12,020, and the incremental cost was ¥2,056 (Table 3). Thus, from the health system perspective, the cost to prevent a case of macrosomia/LGA was ¥30,032/¥26,278, calculated by multiplying NNT by the incremental cost. From the societal perspective, the corresponding cost-effectiveness ratio was ¥32,896/¥28,784 per prevented case of macrosomia/LGA (Table 4).

**Table 4:** Effectiveness and cost-effectiveness of a lifestyle intervention program for women with gestational diabetes in China.

	Effectiveness of intervention		Incremental cost (CNY, ¥)		Incremental effectiveness, Macrosomia /LGA (NNT)‡	Incremental cost-effectiveness ratio (CNY, ¥)			
	Macrosomia Incidence (%)	LGA† Incidence (%)	Health care system	Society		Health care system		Society	
						Macrosomia	LGA†	Macrosomia	LGA†
**Main analysis**									
Shared care	11.2	13.0	1,877	2,056	16/14	30,032	26,278	32,896	28,784
Usual care	17.5	19.9	--	--	--	--	--	--	--
**Sensitivity analysis**									
*Including all 936 women*									
Shared care	12.5	14.0	1,701	1,925	32/20	54,432	34,020	*61*,*600*	*38*,*500*
Usual care	15.6	18.9	--	--	--	--	--	--	--
*Doubling the intervention cost*									
Shared care	11.2	13.0	2,840	3,019	16/14	45,440	39,760	48,304	42,266
Usual care	17.5	19.9	--	--	--	--	--	--	--

All costs were reported in Chinese yuan (CNY, ¥) (1 CNY: 0.159 USD, 31 December 2012).

^‡^ NNT, Number needed to treat to prevent one case.

^†^ LGA, Large for gestational age was defined by gender and gestational age-specific 90^th^ percentiles.

In the sensitivity analysis, the NNT to prevent one macrosomia/LGA was 32/20. The incremental cost was ¥1,701 and the ICER was ¥54,432/¥34,020 for macrosomia/LGA from the health system perspective. The corresponding estimates from the societal perspective were ¥1,925 for the incremental cost and ¥61,600/¥38,500 for the ICER (Table 4). Doubling the intervention led to a higher incremental cost and ICERs (Table 4).

## Discussion

Intensive lifestyle intervention among women with GDM is effective in improving pregnancy outcomes in China [14], but whether this intervention is cost-effective is unclear. Our study results demonstrate that such intervention costs less than ¥33,000 ($5,247) to prevent one macrosomia or LGA. Whether or not implementing this intervention among all women with GDM in China is an efficient use of health care resources depends on the future health and economic consequences of preventing a case of macrosomia or LGA and ultimately society’s willingness to pay for avoiding such adverse outcomes.

Spending less than ¥33,000 ($5,247) to prevent a macrosomia/LGA infant seems to represent a good value in terms of efficient use of limited health care resources in China or elsewhere in the world. First, macrosomia/LGA has both short-term and long-term adverse health complications. The immediate adverse health effects include preterm birth, higher rates of postpartum hemorrhage, increased risk of caesarean delivery, birth trauma, as well as low 1-min Apgar scores [15, 16]. Macrosomia/LGA also has long-term adverse health risks for the offspring. A prospective study using a population-based sample of 21,315 mother-child pairs in China examined the risk factors and long-term health consequences of macrosomia, and found that macrosomic infants showed an increased susceptibility to being overweight and/or obesity in childhood [17]. Obesity among children is a significant risk factor for the development of insulin resistance in a dose-response manner [18]. Moreover, macrosomia is an independent risk factor for developing metabolic syndrome in childhood, and even type 2 diabetes [19] and hypertension [20] later in life.

The long-term adverse health outcomes associated with macrosomia/LGA lead to high future health costs. A literature review reported that a child with obesity bears an extra lifetime medical cost of $19,000 as compared to a child of normal weight in the USA [21]. Persons with diabetes have substantially higher lifetime medical expenditures. Zhuo X. et al showed that in the United States the excess lifetime medical costs for people with diabetes diagnosed at ages 40, 50, 60, and 65 years were $124,600, $91,200, $53,800, and $35,900, respectively [22]. Preventing macrosomia/LGA may lead to a reduction in those long-term medical costs.

Besides macrosomia and LGA, other maternal and infant clinical outcomes in the SC group were also less prevalent as compared with the UC group, including preterm birth (18/339 vs. 28/361), Apgar score at 1 min<7 (0/339 vs. 7/361), premature rupture of membrane (49/339 vs. 69/361) and more [14]. We did not include these potential health benefits in our cost-effectiveness analysis as they were not statistically significant between the two groups in our trial, possibly due to inadequate statistical power to detect a difference. Adding these potential health benefits to the possible benefit from improvement in lifestyle for the mothers and their offspring could further improve the cost-effectiveness of the lifestyle intervention in this population.

Previous cost-effectiveness analyses showed that intensive management of mild GDM was cost-effective in other countries and populations. Moss et al. conducted a cost-consequence analysis of the ACHOIS Trial and reported that intensive management of mild GDM was more expensive compared to routine care but cost-effective at $2,186/quality adjusted life years (QALY) [23]. Ohno, et al. used a decision analytic model to compare treating vs. not treating mild GDM in the United States and showed that the cost per QALY was $20,412 [24]. We did not measure health outcomes using QALY, thus we cannot compare our study results directly with results from these previous studies.

Our study has some limitations. First, we did not evaluate the cost-effectiveness of the intervention measured in cost per QALY as we did not collect quality–of-life data due to a limited study budget. If the lifestyle intervention could improve the quality of life for women with GDM, the intervention could be more cost-effective. Second, the direct non-medical and indirect cost estimates might be subject to recall bias, as the costs associated with travelling to follow-up visits, changes in diet, time lost due to outpatient services, and morbidity were collected from a questionnaire at 4–6 weeks after delivery. However, as the main cost items were collected from the Pregnant Women Health Records electronic database records of each participant, the effect of this recall bias would be minimal. Finally, our findings may not be generalizable to other low- and high-income countries due to different clinical diabetes management across countries.

In conclusion, our study was one of the first to evaluate the cost-effectiveness of lifestyle intervention in women with GDM. We found that lifestyle intervention cost less than ¥33,000 for prevention of a macrosomia/LGA infant in China. Considering the potential severe long-term health and economic consequences of GDM and macrosomia/LGA, intensive lifestyle intervention may be an efficient use of health care resources in China, or possibly in other developing countries.

## Supporting information

S1 Fig Study flow chart of a lifestyle intervention for women with gestational diabetes in Tianjin, China. (DOCX)

S1 Table Baseline clinical and biochemical characteristics of study participants, by assignment. Abbreviations: BMI, body mass index; BP, blood pressure; GDM, gestational diabetes mellitus; GCT, glucose challenge test; HbA1c, hemoglobin A1c; OGTT, oral glucose tolerance test; PG, plasma glucose. † P values were derived from Chi-square Test, Fisher’s Exact Test, or Student T Test unless otherwise specified. ‡ Data were reported as median (interquartile range) and P values were derived from Wilcoxon Two-Sample Test.
(DOCX)

S2 Table Costs associated with routine surveillance, obstetric and neonatal complications due to gestational diabetes, per study participant, by study arm. All costs were reported in Chinese yuan (CNY, ¥) (1 CNY: 0.159 USD, 31 December 2012). MI, Maternity insurance. IP, Individual payment.
(DOCX)

## References

[1] Yang X, Hsu-Hage B, Zhang H, Zhang C, Zhang Y. Women with impaired glucose tolerance during pregnancy have significantly poor pregnancy outcomes. Diabetes care 2002; 25(9):1619–24. 10.2337/diacare.25.9.1619 12196437

[2] Rosenberg TJ, Garbers S, Lipkind H, Chiasson MA. Maternal obesity and diabetes as risk factors for adverse pregnancy outcomes: differences among 4 racial/ethnic groups. American journal of public health 2005; 95(9):1545–51. 10.2105/AJPH.2005.065680 16118366 PMC1449396

[3] Weiner CP. Effect of varying degrees of "normal" glucose metabolism on maternal and perinatal outcome. American journal of obstetrics and gynecology 1988; 159(4):862–70. 10.1016/s0002-9378(88)80156-x 3177539

[4] Hod M, Merlob P, Friedman S, Schoenfeld A, Ovadia J. Gestational diabetes mellitus. A survey of perinatal complications in the 1980s. Diabetes 1991; 40 Suppl 2:74–8. 1748270 10.2337/diab.40.2.s74

[5] Maresh M, Beard RW, Bray CS, Elkeles RS, Wadsworth J. Factors predisposing to and outcome of gestational diabetes. Obstetrics and gynecology 1989; 74(3 Pt 1):342–6. 2761910

[6] Whitaker RC, Pepe MS, Seidel KD, Wright JA, Knopp RH. Gestational diabetes and the risk of offspring obesity. Pediatrics 1998; 101(2):E9 10.1542/peds.101.2.e9 9445519

[7] Feig DS, Zinman B, Wang X, Hux JE. Risk of development of diabetes mellitus after diagnosis of gestational diabetes. CMAJ: Canadian Medical Association journal = journal de l'Association medicale canadienne 2008; 179(3):229–34. 10.1503/cmaj.080012 18663202 PMC2474881

[8] Song C, Lyu Y, Li C, Liu P, Li J, Ma RC, et al Long-term risk of diabetes in women at varying durations after gestational diabetes: a systematic review and meta-analysis with more than 2 million women. Obesity reviews: an official journal of the International Association for the Study of Obesity 2018; 19(3):421–29. 10.1111/obr.12645 29266655

[9] Crowther CA, Hiller JE, Moss JR, McPhee AJ, Jeffries WS, Robinson JS. Effect of treatment of gestational diabetes mellitus on pregnancy outcomes. The New England journal of medicine 2005; 352(24):2477–86. 10.1056/NEJMoa042973 15951574

[10] Landon MB, Spong CY, Thom E, Carpenter MW, Ramin SM, Casey B, et al A multicenter, randomized trial of treatment for mild gestational diabetes. The New England journal of medicine 2009; 361(14):1339–48. 10.1056/NEJMoa0902430 19797280 PMC2804874

[11] Alberti KG, Zimmet PZ. Definition, diagnosis and classification of diabetes mellitus and its complications. Part 1: diagnosis and classification of diabetes mellitus provisional report of a WHO consultation. Diabetic medicine: a journal of the British Diabetic Association 1998; 15(7):539–53. 9686693 10.1002/(SICI)1096-9136(199807)15:7<539::AID-DIA668>3.0.CO;2-S

[12] Metzger BE, Coustan DR. Summary and recommendations of the Fourth International Workshop-Conference on Gestational Diabetes Mellitus. The Organizing Committee. Diabetes care 1998; 21 Suppl 2:B161–7. 9704245

[13] International Association of Diabetes and Pregnancy Study Groups Consensus Panel, Metzger B, Gabbe S, Persson B, Buchanan T, Catalano P, et al International association of diabetes and pregnancy study groups recommendations on the diagnosis and classification of hyperglycemia in pregnancy. Diabetes care 2010; 33:676–82. 10.2337/dc09-1848 20190296 PMC2827530

[14] Yang X, Tian H, Zhang F, Zhang C, Li Y, Leng J, et al A randomised translational trial of lifestyle intervention using a 3-tier shared care approach on pregnancy outcomes in Chinese women with gestational diabetes mellitus but without diabetes. J Transl Med 2014; 12:290 10.1186/s12967-014-0290-2 25349017 PMC4213554

[15] Henriksen T. The macrosomic fetus: a challenge in current obstetrics. Acta obstetricia et gynecologica Scandinavica 2008; 87(2):134–45. 10.1080/00016340801899289 18231880

[16] Jastrow N, Roberge S, Gauthier RJ, Laroche L, Duperron L, Brassard N, et al Effect of birth weight on adverse obstetric outcomes in vaginal birth after cesarean delivery. Obstetrics and gynecology 2010; 115(2 Pt 1):338–43. 10.1097/AOG.0b013e3181c915da 20093908

[17] Gu S, An X, Fang L, Zhang X, Zhang C, Wang J, et al Risk factors and long-term health consequences of macrosomia: a prospective study in Jiangsu Province, China. Journal of biomedical research 2012; 26(4):235–40. 10.7555/JBR.26.20120037 23554754 PMC3596738

[18] Young-Hyman D, Schlundt DG, Herman L, De Luca F, Counts D. Evaluation of the insulin resistance syndrome in 5- to 10-year-old overweight/obese African-American children. Diabetes care 2001; 24(8):1359–64. 10.2337/diacare.24.8.1359 11473070

[19] Wei J, Sung F, Li C, Chang C, Lin R, Lin C, et al Low birth weight and high birth weight infants are both at an increased risk to have type 2 diabetes among schoolchildren in taiwan. Diabetes care 2003; 26:343–8. 10.2337/diacare.26.2.343 12547860

[20] Ruan Y, Mo M, Joss-Moore L, Li YY, Yang QD, Shi L, et al Increased waist circumference and prevalence of type 2 diabetes and hypertension in Chinese adults: two population-based cross-sectional surveys in Shanghai, China. BMJ open 2013; 3(10):e003408 10.1136/bmjopen-2013-003408 24165029 PMC3816232

[21] Finkelstein EA, Graham WC, Malhotra R. Lifetime direct medical costs of childhood obesity. Pediatrics 2014; 133(5):854–62. 10.1542/peds.2014-0063 24709935

[22] Zhuo X, Zhang P, Barker L, Albright A, Thompson TJ, Gregg E. The lifetime cost of diabetes and its implications for diabetes prevention. Diabetes care 2014; 37(9):2557–64. 10.2337/dc13-2484 25147254

[23] Moss J, Crowther C, Hiller J, Willson K, Robinson J, Group. ACISiPW. Costs and consequences of treatment for mild gestational diabetes mellitus—evaluation from the ACHOIS randomised trial. BMC Pregnancy Childbirth 2007; 7:27 10.1186/1471-2393-7-27 17963528 PMC2241640

[24] Ohno M, Sparks T, Cheng Y, Caughey A. Treating mild gestational diabetes mellitus: a cost-effectiveness analysis. American journal of obstetrics and gynecology 2011; 205:282.e1–7. 22071065 10.1016/j.ajog.2011.06.051 PMC3443977

