# Supplementary material for: Within-trial cost-effectiveness of lifestyle intervention using a 3-tier shared care approach for pregnancy outcomes in Chinese women with gestational diabetes
Source: PLoS One. 2020 Aug 20;15(8):e0237738. doi: 10.1371/journal.pone.0237738 (PMC7444483; doi:10.1371/journal.pone.0237738)
Supplement: S1 Fig — (DOCX) [file pone.0237738.s001.docx]

S1 Fig. Study flow chart of a lifestyle intervention for women with gestational diabetes in Tianjin, China

Screened for gestational diabetes (n=19,847)


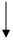


Potential eligible women (n=1388)

Excluded due to:

♦ Not meeting inclusion criteria (n=174)

Diagnosed with diabetes (n=122)

Non-singleton pregnancy (n=20)

Blood type incompatibility (n=1)

Maternal problems (n=31)

♦Refusal (n=222)

♦Error/s in randomisation on the day (n=44)

Randomized (n=948)


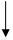


Assigned to shared care (n=474)

Assigned to usual care (n=474)

Excluded those with possible data contamination (n=242)

Study participants with valid data (n=344)

Study participants with valid data (n= 362)

Lost to follow-up due to delivery at non-study hospital (n=5)

Lost to follow-up due to delivery at non-study hospital (n=1)

Final main analysis (n=361)

Final main analysis (n=339)
