# Supplementary material for: Within-trial cost-effectiveness of lifestyle intervention using a 3-tier shared care approach for pregnancy outcomes in Chinese women with gestational diabetes
Source: PLoS One. 2020 Aug 20;15(8):e0237738. doi: 10.1371/journal.pone.0237738 (PMC7444483; doi:10.1371/journal.pone.0237738)
Supplement: S1 Table — Abbreviations: BMI, body mass index; BP, blood pressure; GDM, gestational diabetes mellitus; GCT, glucose challenge test; HbA1c, hemoglobin A1c; OGTT, oral glucose tolerance test; PG, plasma glucose. † P values were derived from Chi-square Test, Fisher’s Exact Test, or Student T Test unless otherwise specified. ‡ Data were reported as median (interquartile range) and P values were derived from Wilcoxon Two-Sample Test. (DOCX) [file pone.0237738.s002.docx]

S1 Table. Baseline clinical and biochemical characteristics of study participants, by assignment

| Variables | Usual Care (n=361) | Shared Care (n=339) |  |
| --- | --- | --- | --- |
|  | Mean (SD)  or n (%) | Mean (SD)  or n (%) | P value† |
| **At the 1^st^ antenatal care visit** |  |  |  |
| Age, year | 29.7(3.2) | 29.9(3.5) | 0.475 |
| Ethnicity |  |  |  |
| Han | 350(97.0%) | 329(97.0%) | 0.940 |
| Others | 11(3.1%) | 10(3.0%) |  |
| Smoking status |  |  | 0.753 |
| Non-smoker | 342(94.8%) | 323(5.3%) |  |
| Ex-smoker | 16(4.4%) | 12(3.5%) |  |
| Current smoker | 3(0.8%) | 4(1.2%) |  |
| Alcohol intake |  |  | 0.507 |
| Non-drinker | 250(69.2) | 244(72.0%) |  |
| Ex-occasional drinker | 97(26.9%) | 79(23.3%) |  |
| Current occasional drinker | 14(3.9%) | 16(4.7%) |  |
| Body height, cm | 163(4.5) | 162(5.1) | 0.091 |
| Pre-pregnancy BMI, kg/m^2^ | 23.4(3.9) | 22.9(3.6) | 0.111 |
| BMI classification |  |  | 0.365 |
| Below 18.5 (underweight) | 23(6.4%) | 25(7.4%) |  |
| 18.0-23.9 (normal weight) | 202(56.0%) | 206(60.8%) |  |
| 24.0-27.9 (overweight) | 91(25.2%) | 77(22.7%) |  |
| 28.0 and above (obesity) | 45(12.5%) | 31(9.1%) |  |
| Systolic BP, mmHg | 107(10.2) | 108(10.9) | 0.742 |
| Diastolic BP, mmHg | 69(7.5) | 70(7.8) | 0.339 |
| Gestational age at the first visit, weeks | 10.8(2.3) | 10.8(2.4) | 0.593 |
| **At screening for and diagnosis of GDM** |  |  |  |
| GCT, mmol/L‡ | 8.9(8.3-9.8) | 9.0(8.4-9.8) | 0.364 |
| Systolic BP, mmHg | 109(10.1) | 109(10.7) | 0.786 |
| Diastolic BP, mmHg | 69(7.6) | 70(7.5) | 0.554 |
| HbA1c, % | 5.0(0.5) | 5.0(0.5) | 0.771 |
| OGTT |  |  |  |
| Fasting PG, mmol/L | 5.0(0.5) | 5.1(0.6) | 0.084 |
| 1-h PG, mmol/L | 10.0(1.3) | 10.1(1.4) | 0.366 |
| 2-PG, mmol/L | 8.4(1.4) | 8.4(1.2) | 0.983 |
| Fasting insulin, mIU/L‡ | 8.7(5.7-12.8) | 9.0(5.6-12.8) | 0.867 |
| 2-h insulin, mIU/L‡ | 86.1(51.1-128.8) | 82.6(50.9-123.1) | 0.703 |
| Weight gain at OGTT, kg | 9.7(4.4) | 9.8(4.4) | 0.774 |
| Gestational age at OGTT, weeks‡ | 26.3(25.4-27.1) | 26.3(25.4-27.4) | 0.410 |

Abbreviations: BMI, body mass index; BP, blood pressure; GDM, gestational diabetes mellitus; GCT, glucose challenge test; HbA1c, hemoglobin A1c; OGTT, oral glucose tolerance test; PG, plasma glucose.

† P values were derived from Chi-square Test, Fisher’s Exact Test, or Student T Test unless otherwise specified.

‡ Data were reported as median (interquartile range) and P values were derived from Wilcoxon Two-Sample Test.
