# Supplementary material for: Within-trial cost-effectiveness of lifestyle intervention using a 3-tier shared care approach for pregnancy outcomes in Chinese women with gestational diabetes
Source: PLoS One. 2020 Aug 20;15(8):e0237738. doi: 10.1371/journal.pone.0237738 (PMC7444483; doi:10.1371/journal.pone.0237738)
Supplement: S2 Table — All costs were reported in Chinese yuan (CNY, ¥) (1 CNY: 0.159 USD, 31 December 2012). MI, Maternity insurance. IP, Individual payment. (DOCX) [file pone.0237738.s003.docx]

S2 Table．Costs associated with routine surveillance, obstetric and neonatal complications due to gestational diabetes, per study participant, by study arm

|  | Shared care (n=339, CNY, ¥) | | Usual care (n=361 CNY, ¥,) | | Difference  (CNY, ¥,) | Data sources | |  |
| --- | --- | --- | --- | --- | --- | --- | --- | --- |
|  |  |  |  |  |  |  | |  |
| Review of hyperglycemia one week after diagnosis of GDM | | 48 | 48 | | 0 | | MI and IP | |
| Multidisciplinary clinical management | |  |  |  | |  | | |
| Primary hospital | | 110 | 120 | -10 | | MI and IP | | |
| Secondary hospital | | 270 | 330 | -60 | | MI and IP | | |
| Tertiary hospital | | 390 | 340 | 50 | | MI and IP | | |
| Hospitalization cost before delivery | | 76 | 59 | 17 | | MI and IP | | |
| Hospitalization cost for delivery | | 8,655 | 7,737 | 918 | | MI and IP | | |
| Total | | 9,549 | 8,634 | 915 | | MI and IP | | |

All costs were reported in Chinese yuan (CNY, ¥) (1 CNY: 0.159 USD, 31 December 2012).

MI, Maternity insurance. IP, Individual payment.
