# Supplementary material for: Within-trial cost-effectiveness of lifestyle intervention using a 3-tier shared care approach for pregnancy outcomes in Chinese women with gestational diabetes
Source: PLoS One. 2020 Aug 20;15(8):e0237738. doi: 10.1371/journal.pone.0237738 (PMC7444483; doi:10.1371/journal.pone.0237738)
Supplement: Attachment — Submitted filename: Response to Reviewers.docx [file pone.0237738.s004.docx]

PONE-D-20-12277

**Response to Reviewer** #**1**

Reviewer #1: Major comment:

1. Could you provide more details about how you randomly assigned the women into SC or UC group? Or provide some statistics to show no other factors would cause the differences regarding the pregnancy outcomes between two groups.

**Response:** Thank you for your comment. Details of the randomization were described in our previous publication. Following the suggestion, we now added more details of randomization as follows: “*A simple randomization procedure without replacement (i.e., by the time sequence of visits to the clinic and a list of priori computer-generated random assignment status by X.Y.) was used to perform the random assignment by the intervention team members.”* We also added a sentence to show no significant differences regarding the pregnancy outcomes between two groups as “*There were no significant differences between the two groups for the baseline clinical and biochemical characteristics.*”.

Minor comment:

1. Some numbers in table 3 are not correct.

**Response:** Thank you for the careful reading . We have corrected the error. .

2. What are the p-values of : macrosomia [the SC group vs. the UC group: 11.2% (38/339) vs. 17.5% (63/361)] and LGA [the SC group vs. the UC group: 13.0% (44/339) vs. 19.9% (72/361)]

**Response:** We have added the p values in the first paragraph of the results.

**Response to Reviewer** #**2**

Reviewer #2: Based on the data from one of the biggest city in CHINA, the authors evaluated the cost-effectiveness of lifestyle intervention in women with GDM and found that lifestyle intervention cost less than ¥33,000 for prevention of a macrosomia/LGA infant in China, so intensive lifestyle intervention may be an efficient use of health care resources in China. Although this paper provides potentially interesting new observations, there are some significant issues that need to be addressed for it to be suitable for publication.

Major point:

1. Only 339 from the SC and 361 from UC are availabel finally, its a very important information , should be shown in the abstract.

**Response:** Thank you for your comment. We have added this information in the abstract.

2. The p value need to be shown in the first paragraph of the results between different groups, without that how to say "the SC significantly reduced the rates of macrosomia....."

**Response:** Thank you for your comment. We have added the p values in the first paragraph of the results.

Minor point:

3. The unit in the sentence ‘35 kcal/day/kg for one kilogram body weight per day for those with BMI at <18.5 kg/m^2^; 30–35 kcal/day for those with BMI at 18.5-23.9 kg/m^2^;

25-30 kcal/day/kg for those with BMI at 24.0-27.9 kg/m2; 25 kcal/day/kg for those

BMI at 28.0 kg/m^2^ and more” is not clear.

**Response:** Thank you for your comment. We have revised this sentence.

Now it reads as “35 kcal per day for one kilogram of body weight (35 kcal/day/kg) for women with BMI at <18.5 kg/m^2^; 30–35 kcal/day/kg for women with BMI at 18.5-23.9 kg/m^2^; 25-30 kcal/day/kg for women with BMI at 24.0-27.9 kg/m^2^; 25 kcal/day/kg for women with BMI at 28.0 kg/m^2^ and more”

4. macrosomia should be a special kind of LGA, so the defination of LGA need to exclude macrosomia.

**Response:** We thank the reviewer for this insight. We agree that macrosomia is a special kind of LGA. However, in our original RCT design, we used the two outcomes as sperate outcome measures for the trial. We defined LGA as birth weight above the gender- and gestational age- specific 90^th^ percentiles using the Tianjin local references, while macrosomia as birth weight ≥4,000 gram. The two measures were both prior defined primary endpoints for our RCT. We reported the effectiveness of intervention using both endpoints in our previous published main analysis. So we preferred not to exclude macrosomia from LGA to keep consistency with the main report.

5. the language of this manuscript need to be improved.

**Response:** Thank you for your comment. Now, a native English editor has copyedited the paper.
